# Supplementary material for: Cultural Divergence in Psychedelic Use among Medical Students: An ESPAD-Adapted Survey among Poles and Iraqis
Source: Eur J Investig Health Psychol Educ. 2024 Mar 5;14(3):563–83. doi: 10.3390/ejihpe14030038 (PMC10968917; doi:10.3390/ejihpe14030038)
Supplement: Supplementary file 1 [file ejihpe-14-00038-s001.zip › ejihpe-2802603-supplementary.pdf]

Table S1. The English version of the ESPAD-adapted survey.

1. What is your sex?
   □ Male
   □ Female
2. What is your age?
   □ Adolescent (10-19)
   □ Young adult (20-39)
   □ Middle-aged (40-60)
3. Concerning your religious affiliations, what is your status?
   □ I believe in my religion, and I practice it
   □ I believe in my religion, but I do not practice it
   □ I do not believe in my religion, but I practice it
   □ I do not believe in my religion, and I do not practice it
4. What is the financial income status of your family?
   □ Low income
   □ Middle income
   □ High income
5. What is your Nationality **(text box)**?
6. Where do you live?
   □ Urban area
   □ Rural area
7. Which of the following best describes your academic performance at the end of the last term?
   □ High
   □ Average
   □ Low
8. During the LAST 30 DAYS, how many days have you missed one or more classes?

	None	1 day	2 days	3-4 days	5-6 days	≥7 days
Because of illness						
Because you skipped						
For other reasons						

9. How often do you do each of the following?

	Never	A few times a year	Once or twice a month	At least once a week	Almost every day
Play computer and video games					
Actively participate in sports, athletics, or exercising					
Read books for enjoyment (do not count schoolbooks)					
Go out in the evening (to a disco, cafe, party, etc.)					
Other hobbies (playing an instrument, singing, drawing, writing)					
Go around with friends to shopping centers, parks, etc.					
Use the internet for leisure activities (chats, music, games, social networks, videos, etc.)					
Play on slot machines (the kind in which you may win money)					

10. What is the highest level of education your father completed?
 □ Completed primary school or less
 □ Completed secondary school
 □ Completed college or university
 □ Don't know

11. What is the highest level of education your mother completed?
 □ Completed primary school or less
 □ Completed secondary school
 □ Completed college or university
 □ Don't know

12. How often do the following statements apply to you?

	Almost always	Often	Sometimes	Seldom	Almost never
My parent(s) set definite rules about what I can do at home					
My parent(s) set definite rules about what I can do outside the home					
My parent(s) know whom I am with during the evenings					
My parent(s) know where I am during the evenings					
I can easily borrow money from my mother and/or father					
I can easily get money as a gift from my mother and/or father					

13. Indicate how you feel about each statement.

	Very strongly disagree	2	3	4	5	6	Very strongly agree
My family really tries to help me							
I get the emotional help and support I need from my family							
I can talk about my problems with my family							
My family is willing to help me make decisions							

14. Does your parents or family know where you spend your weekend nights?
 □ Know always
 □ Know quite often
 □ Know sometimes
 □ Usually don't know

15. How satisfied are you usually with …

	Very satisfied	Satisfied	Neither nor	Not so satisfied	Not at all satisfied	There is no such person
Your relationship with your mother?						
Your relationship with your father?						
Your relationship with your friends?						

16. Do you smoke cigarettes or tobacco?
 □ Yes
 □ No

17. Do you drink alcohol or alcoholic beverages?
□ No
□ Seldom
□ Sometimes
□ Often
18. Have you heard about psychedelics, also known as hallucinogens and entheogens?
□ Yes
□ No
19. How would you define psychedelics **(single answer)**?
□ Psychedelics are CNS depressants and cannot be prescribed for individuals with depression
□ Psychedelics are psychoactive substances that alter consciousness and cognitive processes
□ Psychedelics are anti-inflammatory agents used for treating medical conditions
□ Psychedelics are principally used in general anesthesia and for treating epilepsy
20. Which of the following are psychedelics **(multiple answers)**?
□ Psilocybin
□ Morphine
□ Tramadol
□ Lysergic acid diethylamide (LSD)
□ Dopamine
□ Amphetamine
□ Ayahuasca
□ Paracetamol
□ Dimethyltryptamine (DMT)
21. Have you ever used psychedelics?
□ Never
□ Yes, in the last 30 days
□ Yes, in the last 12 months
□ Yes, more than 12 months ago
22. Why did you try psychedelics for the first time?
□ I have never tried psychedelics
□ Out of curiosity
□ Because my friends/other people offered psychedelics to me
□ To relieve stress
□ Other reasons
23. How difficult do you think it would be for you to get psychedelics if you wanted?
□ Impossible
□ Very difficult
□ Fairly difficult
□ Fairly easy
□ Very easy
□ Don't know
24. How difficult do you think it would be for you to get cannabis if you wanted?
□ Impossible
□ Very difficult
□ Fairly difficult
□ Fairly easy
□ Very easy
□ Don't know
25. In your opinion, what is the level of risk regarding the

	No risk	Low risk	Moderate risk	High risk	Don't know
Use of cannabis once or twice					

Use of cannabis occasionally					
Use of cannabis regularly					
Use of psychedelics once or twice					
Use of psychedelics occasionally					
Use of psychedelics regularly					

26. Have you ever used cannabis or psychedelics to improve your academic performance?
    □ No
    □ Yes
27. What is your own opinion on psychedelics **(text box)**?
28. Which of these adverse effects of psychedelics are you aware of **(multiple answers & text box)**?
    □ Dilated pupils and photosensitivity
    □ Dermatitis (eczema)
    □ Increased heart rate and blood pressure
    □ Motor coordination problems
    □ Venous leg ulcers
    □ Gastrointestinal upset, including nausea and vomiting
    □ Trigeminal neuralgia
    □ Respiratory arrest
    □ Hemorraghic fever
    □ Loss of consciousness or coma
    □ Disseminated intravascular coagulation
    □ Drug-induced psychosis
    □ Septic shock
    □ Hallucinogen-Persisting Perception Disorder
    □ Others **(text box)**

Q29–Q31 ARE ONLY FOR CANNABIS OR PSYCHEDELICS USERS

29. On how many occasions have you used psychedelics?

	A Few times	Sometimes	Frequently
In your lifetime			
During the last 12 months			
During the last 30 days			

30. On how many occasions have you used cannabis?

	A Few times	Sometimes	Frequently
In your lifetime			
During the last 12 months			
During the last 30 days			

31. If you ever used cannabis or any psychedelics, where did you get the substance(s) from?
    □ Offered by a family member
    □ By a street dealer
    □ Through the Internet
    □ From a friend or a distant acquaintance
    □ By courier or mail
    □ Others

Table S2. Socio-demographic and socio-cultural differences between Poles and Iraqis.

Variable (Survey Question)			p-value	Odds Ratio	95% Confidence Interval	
Age	Iraq/Poland	Junior Students	<0.001	6.45	3.92	10.61
		Senior Students				
Religious Stance	Iraq/Poland	Present	<0.001	18.01	12.44	26.08
		Absent				
Residence	Iraq/Poland	Urban	<0.001	1.13	1.06	1.20
		Rural				
Academic performance (in last semester)	Iraq/Poland	Average/Low	<0.001	1.90	1.39	2.60
		High				
Missing classes (because of illness)	Iraq/Poland	Yes	<0.001	3.70	2.59	5.26
		No				
Missing classes (intentional)	Iraq/Poland	Yes	<0.001	5.68	4.07	8.00
		No				
Missing classes (other reasons)	Iraq/Poland	Yes	<0.001	3.62	2.63	5.00
		No				
Playing videogames	Iraq/Poland	Yes	0.015	1.47	1.08	2.01
		No				
Participating in Sports	Poland/Iraq	Yes	<0.001	10.97	6.07	19.82
		No				
Reading cultural books	Poland/Iraq	Yes	<0.001	5.76	3.49	9.49
		No				
Going out in the evening	Poland/Iraq	Yes	<0.001	5.87	3.14	10.98
		No				
Playing an instrument (or other hobbies)	Poland/Iraq	Yes	<0.001	4.51	3.11	6.53
		No				
Going around (with friends)	Poland/Iraq	Yes	<0.001	3.20	1.63	6.30
		No				
Using the internet (for leisure activities)	Poland/Iraq	Yes	0.001	13.92	1.85	104.843
		No				
Father's educational level	Poland/Iraq	>=Secondary school	0.028	2.24	1.08	4.67
		<=Primary school				
Mother's educational level	Poland/Iraq	>=Secondary school	<0.001	5.88	2.63	13.16
		<=Primary school				
Parents set definite rules (outside home)	Iraq/Poland	Yes	<0.001	6.17	4.46	8.48
		No				
Parents know (whom I am with in the evenings)	Iraq/Poland	Yes	<0.001	5.35	3.27	8.77
		No				
Parents know (where I am in the evenings)	Iraq/Poland	Yes	<0.001	5.08	3.15	8.20
		No				
I can easily borrow money (from parents)	Iraq/Poland	Yes	0.007	2.11	1.22	3.66
		No				
I get emotional support I need (from family)	Iraq/Poland	Yes	0.001	1.73	1.26	2.37
		No				
My family helps me make decisions	Iraq/Poland	Yes	0.001	1.73	1.26	2.37
		No				
Parents know (where I spend weekend nights)	Iraq/Poland	Yes	<0.001	6.63	3.47	12.66
		No				
Satisfaction (relationship with mother)	Iraq/Poland	Yes	0.007	1.64	1.14	2.34
		No				
Satisfaction (relationship with father)	Iraq/Poland	Yes	<0.001	3.14	2.27	4.35
		No				

Satisfaction (relationship with friends)	Poland/Iraq	Yes	0.003	1.78	1.21	2.62
		No				
Smoking cigarettes or tobacco	Poland/Iraq	Yes	<0.001	2.45	1.68	3.58
		No				
Drinking alcohol	Poland/Iraq	Yes	<0.001	180.44	100.17	325.04
		No				
Have you heard about psychedelics?	Poland/Iraq	Yes	<0.001	7.96	4.75	13.36
		No				
How would you define psychedelics?	Poland/Iraq	Yes	<0.001	42.37	19.56	91.78
		No				
Have you ever used psychedelics?	Poland/Iraq	Yes	<0.001	23.07	9.18	57.99
		No				
Difficulty of getting psychedelics	Poland/Iraq	Easy	<0.001	2.16	1.59	2.94
		Difficult				
Difficulty of getting cannabis	Poland/Iraq	Easy	<0.001	9.08	6.13	13.45
		Difficult				
Cannabis risk of use, once or twice	Poland/Iraq	Low risk	<0.001	4.03	2.89	5.62
		High risk				
Cannabis risk of use, occasionally	Poland/Iraq	Low risk	<0.001	2.47	1.83	3.33
		High risk				
Psychedelics risk of use, once or twice	Poland/Iraq	Low risk	0.054	1.33	0.99	1.79
		High risk				
Psychedelics use as cognitive enhancers	Poland/Iraq	Yes	0.003	12.44	1.57	98.71
		No				
Opinion on psychedelics	Poland/Iraq	Positive	0.006	1.51	1.13	2.02
		Negative				
